# Mr. Balfour on Medical Science

**Published:** 1901-06-01

**Authors:** 


### The Hospital

A Journal of

The Medical Sciences and Hospital Administration.

Vol. XXX.—No. 766.] Edited by Sir Henry Burdett, K.C.B., and Solomon C. Smith, M.D., M.R.C.P. [June 1, 1901.

### Mr. Balfour on Medical Science.

Without entirely agreeing with some of the embroidery with which Mr. Balfour decorated his interesting speech on the occasion of the festival dinner of the Medical Graduates' College and Polyclinic, we may at least admit that he made out a very good case. He showed how great are the claims of the London Polyclinic upon the sympathy of those who have at heart the welfare of mankind, who wish to improve the art of easing suffering and beating off disease, and who desire that Englishmen, not in England only, but in our most distant dependencies, should have the benefit of the latest medical discoveries.

He also showed how deficient London has hitherto been in the machinery for post-graduate study, pointing out how difficult it must always be, except with the aid of some such institution as the Polyclinic, for practitioners to keep abreast of medical progress; and then he depicted in forcible colours the ignominious position into which, in comparison with other nations, England has been content to drift in regard to the teaching of science, in consequence apparently of our disinclination—a disinclination displayed both by governments and millionaires—to cast our bread upon the waters in the shape of endowments for scientific work.

The brazen image before which the English nation grovels should be labelled "quick returns." To secure approval, expenditure must now not only pay but pay quickly. Perhaps, as Mr. Balfour suggests, we lack the imagination required to show what can be done for the happiness of mankind by apparently remote and abstract studies bearing upon medical science, but whatever the explanation the fact remains that, so far as concerns equipment for scientific work and scientific education, we have allowed ourselves to be outstripped by other nations. "I do not believe," says Mr. Balfour, "that any man who looks round the equipment of our Universities, or medical schools, or other places of education, can honestly say in his heart that we have done enough to equip research with all the costly armoury which research must have in these modern days. We, the richest country in the world, lag behind Germany, France, Switzerland, and Italy. Is it not disgraceful? Are we too poor or are we too stupid?"

Yes. That is it. Are we too poor or are we too stupid? We are on the horns of a dilemma, and, in view of the evidences of wealth on every side, we are afraid that stupidity has it—stupidity and lack of imagination.

We would point out, moreover, that the present position of science teaching and scientific endowment may well appeal to many who, although they may not perhaps care a rap about science, do at least take a pride in English institutions and in English ways of doing things; for what is just now being weighed in the balance is that peculiarly English institution private enterprise in public work.

It is impossible to regard our few research endowments, our struggling schools, and our lack of appliances for post-graduate study (the latter being the want with which the Polyclinic especially tries to grapple), and to compare our deficiencies in these respects with the plenitude of resources displayed by other nations under a different *régime*, without seeing that the efficiency of private enterprise as a means of meeting public wants is being seriously discredited.

We are proud to say, in this country, that we leave to private endeavour and to private benevolence many duties which in other and less fortunate countries are entrusted to the Government. But in this, as in most things, the seed must be judged by its fruits; and if the policy of private enterprise on which England sets such store is not to be displaced by a policy which is far from being to England's liking, such private enterprise and benevolence must show that it exists and must hold out a hand which shall save England from the foreign custom of trusting all these matters to the Government.

It would be easy to protest, indeed we have heard it said, "This is a very pretty sentiment, my friend, but what has it all got to do with the Polyclinic?" And there's the rub. Mr. Balfour made a good speech, but he made it along the lines of least resistance. After giving the Polyclinic his blessing he threw down the reins and allowed his hobby-horse to amble round the congenial field of endowment of research, which was interesting, although not absolutely *apropos*, for glad as we are to give all praise to those who are responsible for the management of the Polyclinic, we can hardly at present bring ourselves to regard this institution seriously as representative of research.

Its chief *métier* seems to lie in other fields. On the one hand it offers consultant advice to those who are unable to afford ordinary consultant fees, and gives it in a form in which it is not easily obtained elsewhere; and in so doing it well deserves the assistance of the charitable. On the other hand, it offers to men who have already qualified, but who wish to sip again at the fount of knowledge, classes, lectures, and demonstrations arranged specially to meet post-graduate wants, and in so doing it performs a work which must in the end be of great advantage to the public. New conditions bring new requirements. In days when progress was slow one medical education served a life-time. Now the whole gamut of knowledge is set in a different key every few years, and it becomes a matter of the greatest public importance that facilities should be offered to the rank and file of the medical profession for bringing themselves up to date, and becoming acquainted with all that is most recent in medical science. This is an affair for the philanthropist. It would be childish to suggest that all the great things spoken of by Mr. Balfour hinged on the success of this one institution; but at the present moment the London Polyclinic does stand for the idea of post-graduate teaching, an idea the practical embodiment of which will be of the greatest service to mankind, and those who, in addition to wealth, possess intelligence may well feel drawn to lend a helping hand to a work so useful.

